# Effect of Cinnamaldehyde on *C. albicans* cell wall and (1,3)- β – D-glucans in vivo

**DOI:** 10.1186/s12906-021-03468-y

**Published:** 2022-01-31

**Authors:** Jie-Hua Deng, Xiao-Guang Zhang, Gang-Sheng Wang, Jing-Na Luo, Jia Wang, Xiao-Ming Qi, Yan-Ling Li

**Affiliations:** 1 Department of Dermatology and Venereology, Second Hospital of Hebei Medical University, Shijiazhuang, 050000 Hebei China; 2 Translational Medicine Center, Second Hospital of Hebei Medical University, Shijiazhuang, 050000 Hebei China; 3 Quality Control Department, Second Hospital of Hebei Medical University, Shijiazhuang, 050000 Hebei China; 4 Electron Microscopy Room of the Second Affiliated Hospital of Hebei Medical University, Shijiazhuang, 050000 Hebei China

**Keywords:** Cinnamaldehyde, *C. albicans* infection, Fungal cell wall, (1,3)-β-D-glucan, Cell membrane, Electron microscope, Animal model

## Abstract

**Background:**

The incidence rate of invasive candidiasis is high, its treatment is difficult, and the prognosis is poor. In this study, an immunosuppressive mouse model of invasive *Candida albicans (C. albicans)* infection was constructed to observe the effects of cinnamaldehyde (CA) on the *C. albicans* cell wall structure and cell wall (1,3)-β-D-glucan contents. This study provides a theoretical basis for CA treatment to target invasive *C. albicans* infection.

**Methods:**

Immunosuppressed mice with invasive *C. albicans* infection were given an oral dosage of CA (240 mg.kg^− 1^.d^− 1^) for 14 days. Then, mouse lung tissue samples were collected for detection of the levels of (1,3)-β-D-glucan and transmission electron microscopy observations, using fluconazole as a positive control and 2% Tween 80 saline as a negative control.

**Results:**

The immunosuppressive mouse model of invasive *C. albicans* infection was successfully established. The levels of (1,3)-β-D-glucan in the CA treatment group, fluconazole positive control group, invasive *C. albicans* infection immunosuppressive mouse model group, and 2% Tween 80 normal saline control group were 86.55 ± 126.73 pg/ml, 1985.13 ± 203.56 pg/ml, 5930.57 ± 398.67 pg/ml and 83.36 ± 26.35 pg/ml, respectively. Statistically, the CA treatment group, fluconazole positive control group and invasive *C. albicans* infection immunosuppressive mouse model group were compared with each other (*P* < 0.01) and compared with the 2% Tween 80 saline group (*P* < 0.01), showing that the differences were very significant. Comparison of the CA treatment group with the fluconazole positive control group (*P* < 0.05) displayed a difference as well. Electron microscopy showed that CA destroyed the cell wall of *C. albicans*, where the outer layer of the cell wall fell off and became thinner and the nuclei and organelles dissolved, but the cell membrane remained clear and intact.

**Conclusion:**

CA destroys the cell wall structure of *C. albicans* by interfering with the synthesis of (1,3)-β-D-glucan to kill *C. albicans*. However, CA does not affect the cell membrane. This study provides a theoretical basis for CA treatment to target invasive *C. albicans* infection.

## Background

Invasive candidiasis is an infection caused by a yeast, which is a type of fungus, called *Candida*. Invasive candidiasis is a serious infection that affects the blood, heart, brain, eyes, bones and/or other parts of the body [1]. *Candida* usually lives inside the body (in the mouth, throat, intestines, vagina, etc.) and on the skin without causing any problems [2]. However, in some high-risk patients, *Candida* can enter the blood or internal organs and cause infection. *Candida* blood infection (also called candidaemia) is the most common form of invasive candidiasis. In the United States, candidaemia is one of the most common causes of blood infections in hospitalized patients [3, 4], which usually leads to long hospital stays and death. It also causes high medical expenses [5]. Clinically, the target of most antifungal drugs is the fungal cell membrane [6]. For example, the triazole compounds fluconazole, itraconazole, and voriconazole and the polyene amphotericin B all act on ergosterol in the cell membranes of fungi [7]. These drugs inhibit only the growth of fungi and have no fungicidal effects; in addition, these drugs have strong toxicity and side effects and are prone to drug resistance [8–10]. A new class of antifungal drugs, echinocandins (caspofungin, micafungin and anidulafungin), affect the fungal cell wall structure by inhibiting (1,3)-β-D-glucan synthase and can kill fungal cells by destroying the structure of the fungal cell wall [11, 12]. However, to date, the use of targeted antifungal drugs has not been able to meet clinical needs. Since fungal cell walls are unique to fungal cells, necessary for fungal cell survival and do not exist in mammalian cells, new drugs targeting fungal cell walls have become a research hotspot. CA (chemical name 3-phenyl-2-propenal), is a natural alkene group-containing organic compound from the cinnamon plant and is the main active component of the volatile oil of this plant. This light-yellow oily liquid exists as a trans isomer with a chemical formula of C_6_H_5_CHCHCHO and a relative molecular mass of 132.6. A large number of pharmacological studies on CA in China and other countries have shown that it has anti-inflammatory, hypoglycaemic, antitumour, antibacterial and other pharmacological activities [13, 14]; in particular, it has antimicrobial activity against a variety of fungi [15, 16]. However, the current literature reports on CA are all in vitro studies, and there have been no reports on the mechanism of action of CA in vivo. Based on our previous research, CA has good efficacy and safety for the treatment of *C. albicans* infection in mice [17], and this study further explores the mechanism underlying the effects of CA on *Candida* in vivo. The results are reported below.

## Methods

### Materials

#### Experimental strains and animals

The Institute of Microbiology, Chinese Academy of Sciences, provided *C. albicans* (ATCC 90028) and Beijing Weitong Lihua Laboratory Animal Technology Co., Ltd. provided 6- to 8-week-old male BALB/c mice weighing 22–25 g with an animal quality certificate (SPF level, licence number SCXK (Beijing), 2016–0001).

#### Drugs and equipment

CA (purity greater than 98%) was provided by Jiangxi Cedar Medical Oil Co., Ltd. (batch number: 20171002); cyclophosphamide was provided by Jiangsu Hengrui Pharmaceutical Co., Ltd. (batch number: 20170830); fluconazole was provided by Pfizer Pharmaceutical Co., Ltd. (batch number: 20170630); Gram-negative bacteria lipopolysaccharide (LPS) was provided by Zhanjiang Anders Biotechnology Co., Ltd. (batch number: 20170801); Dynamic test tube detector provided by Shenzhen Ryder Power Technology Co., Ltd. (LKM02–32). Microscope, model: Olympus CX43 (10 times X10, 10 times X40); Transmission electron microscope (JEOL JEM-1200EX type); Ultra-clean workbench (Beijing Semiconductor No. 1 Factory).

#### Experimental location

Experiments were performed at the Animal Experiment Center of the Second Hospital of Hebei Medical University. The feeding conditions were as follows: IVR laminar flow cabinet, temperature 22 °C, humidity 50–70%, 5 mice/cage. Protocols were approved by the Animal Care and Use Committee of Hebei Medical University (approval number: HBYD-20170230).

### Experimental grouping

The experimental study of 170 mice is divided as follows using a random number table: (1) immunosuppressive group 40 (including 30 immunosuppressed mice and 10 normal saline control mice); (2) 30 immunosuppressive model mice with invasive *C. albicans* infection (model group) and 10 mice in the normal saline control group; and (3) 90 mice in the CA experimental groups, including 30 immunosuppressive model mice with invasive *C. albicans* infection treated with CA (CA group), 30 mice in the fluconazole positive control group (Fluconazole group), and 30 mice treated with the negative control 2% Tween 80 saline (saline group). The study used a double-blind approach and samples were collected for testing.

#### Immunosuppressive group

Thirty BALB/c mice were injected intraperitoneally with 200 mg.kg^− 1^.d^− 1^ cyclophosphamide for two consecutive days. Blood was collected from the tail 1.0 day before treatment and 2.0, 4.0, 6.0, and 8.0 days after treatment. White blood cells and neutrophils in each sample were counted using a Beckman Coulter Hmx five-stage automatic blood analyser [18]. As a normal saline control group, 10 BALB/c mice were injected intraperitoneally with 0.25 ml of 0.9% normal saline for 14 days.

#### Invasive *C. albicans* infection model group

On the fourth day of immunosuppression, 30 mice were intraperitoneally injected with 0.25 ml of *C. albicans* (1 × 10^7^ cfu/ml). Then, the mice were observed for 14 days. On the day that each mouse died or on the 14th day after treatment, an overdose of pentobarbital (150 mg/kg) was injected intraperitoneally to each mouse to stop the heartbeat and initiate respiratory failure and death. Then, kidney, liver and lung tissue specimens were collected for fungal microscopic examination, culture, pathological examination, (1,3)-β-D-glucan detection and electron microscopic observations [19]. On the fourth day of immunosuppression in 10 mice, 0.25 ml of normal saline was injected intraperitoneally followed by observation for 14 days as a control.

#### CA experimental groups

On the fourth day of immunosuppression, 30 mice were intraperitoneally injected with 0.25 ml of *C. albicans* (1 × 10^7^ cfu/ml) and 12 h later, 240 mg.kg^− 1^.d^− 1^ was orally administered followed by continued administration for 14 consecutive days (human effective dose × 10 = mouse dose), for a total dose of 3360 mg/kg [20]. The total fluconazole dose (240 mg. kg^− 1^.d^− 1^) of the 30 mice in the positive control group over 14 consecutive days was 3360 mg/kg. In the negative control group (not infected with *C. albicans*), 30 mice orally received 0.25 ml of 2% Tween 80 in normal saline for 14 consecutive days. On the 15th day, all surviving mice were intraperitoneally injected with sodium pentobarbital solution (150 mg/kg) and euthanized. Then, a mouse lung tissue specimen (0.5 g) was collected from each mouse under aseptic conditions for (1,3)-β-D-glucan detection and electron microscopy observation.

### Preparation of drugs

Two grams of Tween 80 was added to 97.76 ml of 0.9% saline to prepare a 2% Tween 80 solution.

Then, 240 mg of CA and 240 mg of fluconazole were gradually added to the 2% Tween 80 solution to obtain 0.24% CA and fluconazole solutions, respectively.

### Preparation of a fungal yeast suspension

A *C. albicans* yeast suspension was prepared in 0.1% Tween 80 saline at a concentration of 1 × 10^7^ cfu/ml for later use. Then, the yeast suspension was diluted 10-fold with 0.1% Tween 80 saline, and 100 μl of the diluted solution was inoculated into a glucose protein agar plate. The cells were cultured at 37 °C, and the colonies were counted to determine the survival rate of the *Candida* strain.

### Fungal detection

Lung tissue specimens of the mice in the invasive *Candida* infection model group, CA treatment group and fluconazole control group were prepared with 10% potassium hydroxide, examined under a microscope and cultured on glycoprotein one agar. The agar was observed for the growth of *C. albicans*.

### Histopathological observation

At the end of the experiment, all mice were euthanized by anaesthesia. Then, lung tissue specimens were collected and completely fixed in 4% paraformaldehyde solution. They were conventionally dehydrated, cleared and embedded to make 5 μm thick sections, and the sections were subjected to periodic acid-Schiff (PAS) staining. Histopathological changes were observed under a microscope.

### (1,3)-β-D-glucan detection

Lung tissue specimens of the mice in the *C. albicans* infection model group, CA treatment group and fluconazole control group were collected, and the contents of (1,3)-β-D-glucan in lung tissue were determined by the velocity turbidimetric method. Usually, 500 mg of tissue homogenate was centrifuged at 400×g for 10 min. Then, 100 μg of the supernatant was added to a sample dilution bottle and mixed gently with a vortex, and then the sample was placed in a test tube thermostat (75 °C) for 10 min. Then, a pipette was used to add 0.25 μg of reagent to the LPS reagent to prepare a solution. After 100 μg of the prepared sample test solution was added to the reaction tube, 50 μg of the reagent solution was added. Then, the solutions were mixed, and each test tube was inserted into the LKM dynamic tube tester. Before reading the results, the reaction was carried out at 37 °C for 75 min.

### Transmission electron microscopy (TEM) observation

Lung tissue specimens were collected from mice in the model group, CA group and fluconazole group, fixed with 2% glutaraldehyde and 1% phosphotungstic acid, and dehydrated in an ethanol series (50, 70, 95, 100%) followed by acetone and embedded in epoxy resin. Ultra-thin sections were prepared, stained with uranyl acetate and lead citrate, and observed under a transmission electron microscope.

### Euthanasia of mice

After 14 days, the surviving mice in the model group, CA group and fluconazole group were given an overdose of 1% sodium pentobarbital (150 mg/kg) intraperitoneally. The mice quickly lost consciousness without struggling, their heartbeat and respirations gradually stopped, and the mice eventually died of respiratory failure.

### Statistical analysis

SPSS 21.0 statistical software was used for statistical analysis. The measurement data results are expressed as the mean ± standard deviation and comparison of the count data between groups was performed with the chi-square test. Comparative analysis between groups was performed by analysis of variance, and pairwise comparison of data was performed by the LSD t test. At α = 0.05, all differences were regarded as statistically significant when *P* < 0.05.

## Results

### Establishment of an immunosuppressed BALB/c mouse model

Table 1 shows that BALB/c mice were injected intraperitoneally with 200 mg.kg^− 1^.d^− 1^ cyclophosphamide for 2 consecutive days. The total numbers of white blood cells and granulocytes in the immunosuppressed mice on day 4 were 1.48 × 10^9^/L and 16 × 10^9^/L, respectively, which were significantly lower than those in the normal saline control group (*P* < 0.01). The white blood cell count gradually returned to normal on the 8th day. After 28 days of observation, the mice did not die naturally. These results show that intraperitoneal injection of cyclophosphamide for 2 consecutive days can effectively induce immunosuppression in mice.

**Table 1:** Effect of cyclophosphamide on leucocytes and neutrophils in mice (10^9^ cells L^− 1^)

Day	Immunization group (*n* = 30)		Control group (*n* = 10)	
	Leucocytes	Neutrophils	Leucocytes	Neutrophils
Before treatment	6.82 ± 1.22	58.31 ± 8.29	6.84 ± 1.36	62.81 ± 5.22
After treatment, 2 days	3.09 ± 0.55	32.38 ± 8.26	7.09 ± 1.67	69.27 ± 5.80
After treatment, 4 days	1.48 ± 0.68	16.82 ± 1.20	6.83 ± 1.60	10.35 ± 3.82
After treatment, 8 days	7.68 ± 1.21	62.46 ± 8.29	6.96 ± 1.18	57.35 ± 3.20

Comparison of leucocyte and neutrophil counts between the immunization group and normal saline control group, *P* < 0.01

### Construction of an animal model of *C. albicans* infection in immunosuppressive mice

#### Fungal inspection

This program builds animal models based on literature [19, 20]. On the 4th day of mouse immunosuppression and after intraperitoneal injection of 0.25 ml of 1 × 10^7^ cfu/ml *C. albicans* suspension, 2 mice died on the 2nd day, 6 mice died on the 3rd day, 6 mice died on the 4th day, 2 mice died on the 5th day, 2 mice died on the 6th day, 2 mice died on the 7th day, and 1 mouse died on the 8th day. A total of 21 mice died within 14 days for a mortality rate of 70%. The mice in the saline control group did not die, and there was a significant difference between the two groups (*P* < 0.01) (Table 2).

**Table 2:** Mortality of mice in the invasive *C. albicans* infection model group (%)

Group	n	2 d	3 d	4 d	5 d	6 d	7 d	8 d	9 d	10 d	11 d	12 d	13 d	14 d	Mortality %
Model	30	2	6	6	2	2	2	1	0	0	0	0	0	0	70
Saline	10	0	0	0	0	0	0	0	0	0	0	0	0	0	0

Comparison between the model and control groups using SPSS 21.0 (*P* < 0 .01)

**Fig. 1:**
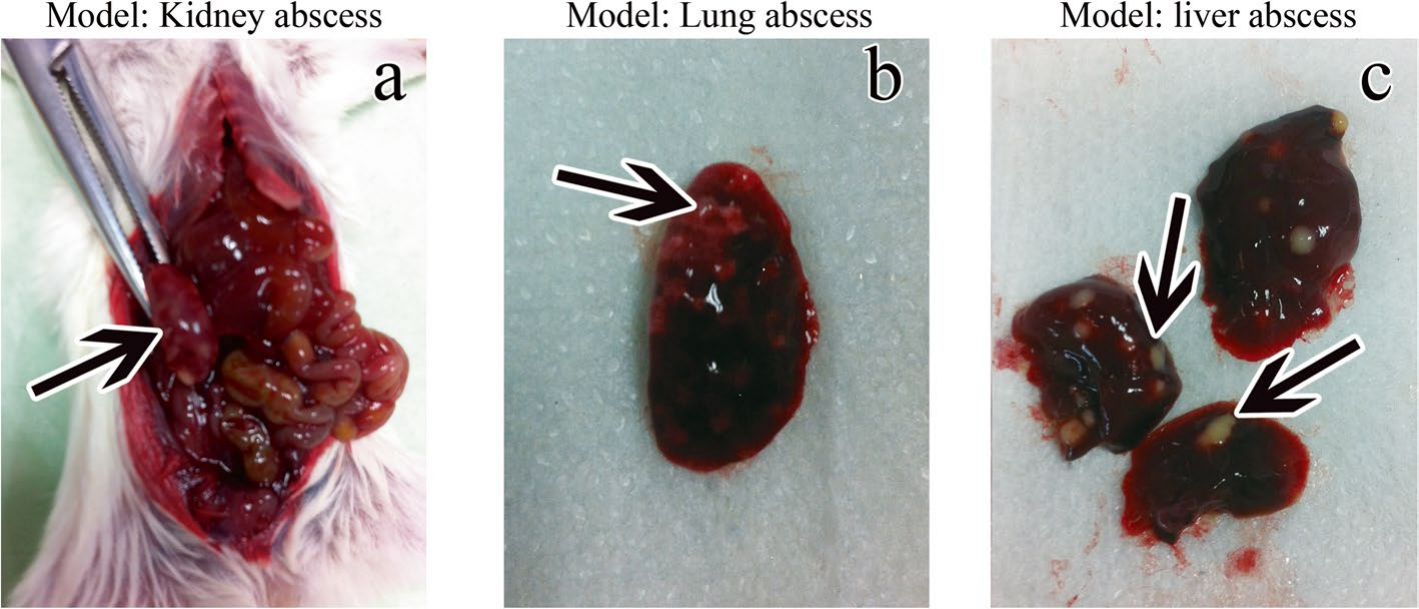
After the immunosuppressive mice died of invasive *C. albicans* infection on the second day, autopsy showed the following. **a** Multiple abscesses in the kidney; **b** Multiple abscesses in the lungs; **c** Multiple abscesses in the liver

**Fig. 2:**
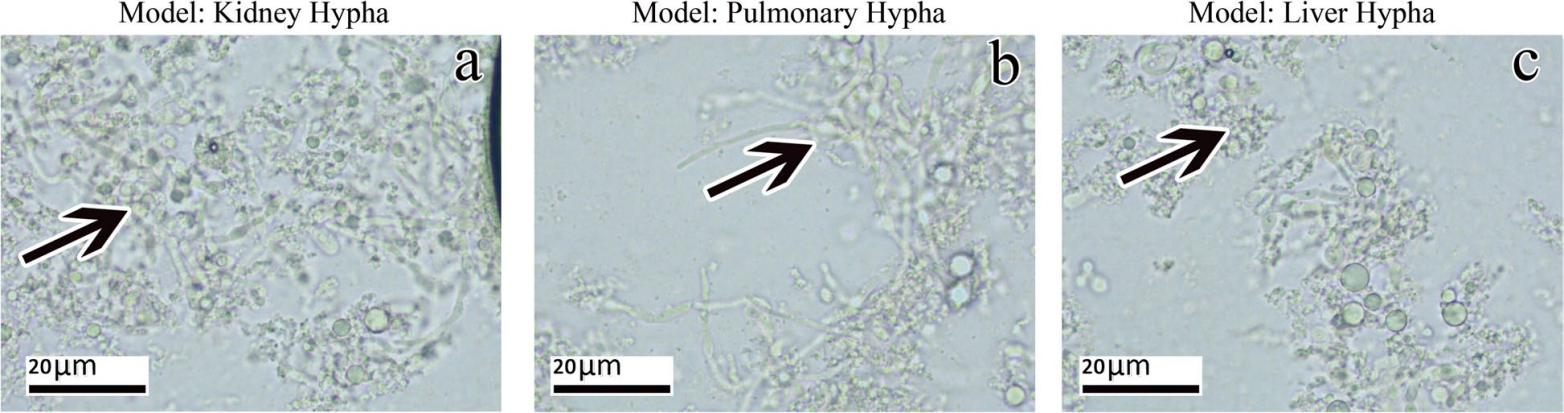
Organ tissue specimens of immunosuppressed mice infected with invasive *C. albicans* on the second day after death were collected and prepared with 10% sodium hydroxide. Microscopy observations showed the following. **a** Most of the hyphae and sprouting yeast in the kidney tissue (40×). **b** Most of the hyphae and sprouting yeast in the lung tissue (40×); **c** Most of the hyphae and sprouting yeast are in the liver tissue (40×)

The dead mice were dissected on days 2, 3, 4, 5, 6, 7, and 8, and multiple abscesses were observed in kidney, lung and liver tissues (Fig. 1a, b, c). Direct microscopic examination revealed a large number of hyphae and yeast in kidney, lung and liver tissues (Fig. 2a, b, c). Kidney, lung and liver tissue specimens all showed growth of *C. albicans.*

#### Histopathological changes

**Fig. 3:**
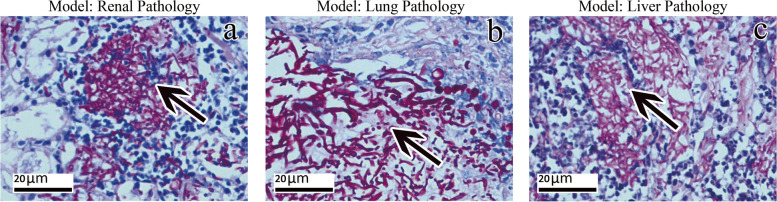
Organ tissue specimens of immunosuppressive mice infected with invasive *C. albicans* were collected on the second day of death after histopathology (PAS staining), and the following was observed under a microscope. **a** Most of the hyphae and budding yeast in the kidney tissue (40×); **b** Most of the hyphae and budding yeast in the lung tissue (40×); **c** Most of the hyphae and budding yeast in the liver tissue (40×)

After the immunosuppressed mice were infected with *C. albicans*, kidney, lung and liver tissue specimens were collected for pathological examination within 2–14 days. After PAS staining, obvious histopathological changes were observed in kidney, lung and liver tissues, where a large number of hyphae and yeast were observed. The normal tissue structure disappeared and was filled with inflammatory exudate, and the infiltration of inflammatory cells (mainly neutrophils) was observed, as shown in (Fig. 3a, b, c).

According to the fungal and pathological examinations, an animal model of invasive *C. albicans* infection in immunosuppressive mice was successfully constructed.

### Effects of CA on (1,3)-β-D-glucan in lung tissue of mice infected with *C. albicans*

The levels of (1,3)-β-D-glucan detected in the CA treatment group, fluconazole positive control group, invasive *C. albicans* infection model group, and 2% Tween 80 normal saline negative control group were 86.55 ± 126.73 (pg/ml), 1985.13 ± 203.56 (pg/ml), 5930.57 ± 398.67 (pg/ml) and 83.36 ± 26.35 (pg/ml), respectively. The values of the CA treatment group and the fluconazole positive control group were compared with those of the invasive *C. albicans* infection model group and the 2% Tween 80 normal saline negative control group and a significant difference was found (*P* < 0.01). Comparing the value in the CA treatment group with that of the fluconazole positive control group, *P* < 0.05, indicating a significant difference (Table 3).

**Table 3:** The effect of CA on (1,3)-β-D-glucan in pulmonary *C. albicans* infection (pg/ml)

Group	n	(1,3)-β-D-glucan	***F***	*P*
CA	30	86.55 ± 126.73	537.86	0.01
Fluconazole	30	1985.13 ± 203.56		
Model	30	5930.57 ± 398.67		
Tuwen80Saline	30	83.36 ± 26.35		

Statistical analysis was conducted with SPSS 21.0 software, comparing the CA group and fluconazole group with the model group (*P* < 0.01) and the Tuwen80 saline negative control group (*P* < 0.01) and comparing the CA group with the fluconazole group (*P* < 0.05)

Explanation: CA has a significant effect on (1,3)-β-D-glucan content in the cell wall of *C. albicans*.

### Effects of CA on the cell structure of *C. albicans*

**Fig. 4:**
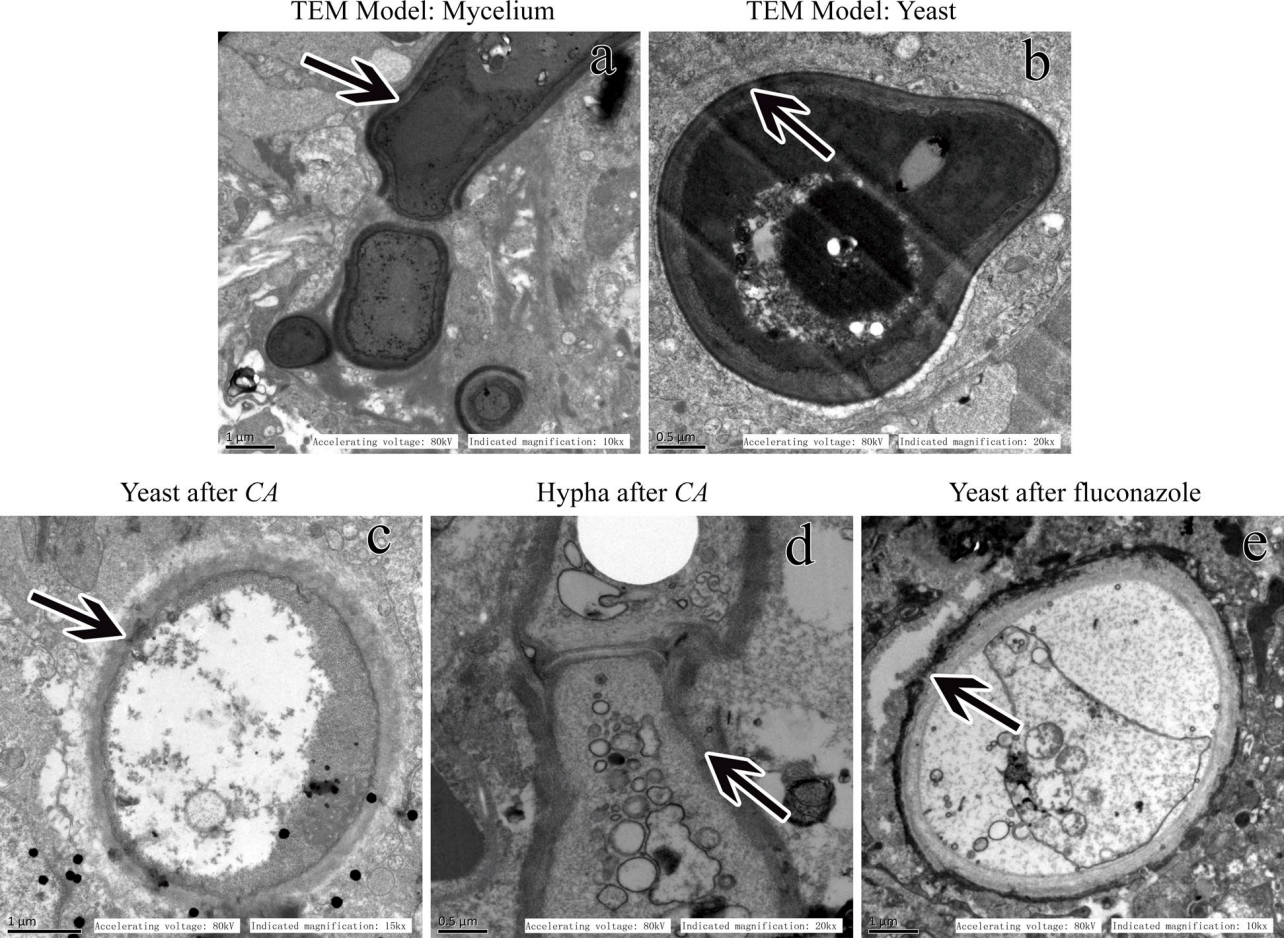
Lung tissue specimens collected from immunosuppressed mice on the second day after death from invasive *C. albicans* infection were prepared for observations by transmission electron microscopy (TEM) and electron microscopy. **a** The mycelial cell wall had a plate-like multilayer thick-walled structure with a clear cell membrane and complete contents. **b** The budding yeast cell wall had a multi-layer thick-walled structure with a clear cell membrane and complete contents. **c** The outer layer of the yeast cell wall of *C. albicans* dissolved and fell off, the cell wall became thinner, and the cell nuclei and organelles dissolved and disappeared. However, the cell membrane was clear and intact. **d** The outer layer of the cell wall of the hyphae of *C. albicans* dissolved and fell off, the cell wall was notably thinned and severely damaged, and the cell nuclei and organelles dissolved and disappeared. **e** After 14 days of fluconazole to treat invasive *C. albicans* infection in immunosuppressive mice, lung tissue specimens were collected and prepared for transmission electron microscopy (TEM). The following were observed under the electron microscope. The yeast cell wall of *C. albicans* was relatively complete, but the cell membrane was completely shed and the cell nuclei and organelles dissolved and disappeared

Transmission electron microscopy observations showed that the yeast and mycelium cell walls of the invasive *C. albicans* in the infection model group were plate-like multilayer thick-walled structures with transparent cell membranes and complete inclusions, as shown in (Fig. 4a, b).

In the CA treatment group, the hyphae of *C. albicans* and the outer layer of the yeast cell wall dissolved and fell off and was notably thinned, the surface was incomplete, and the cell wall was severely damaged. The drug CA directly penetrated the cell membrane and destroyed the nuclei and organelles, causing cell oedema, dissolution, denaturation and necrosis and leading to cell death. However, the cell membranes of the mycelium and yeast were still intact (Fig. 4c, d). The results show that the target of CA in the body is the cell wall of spores and hyphae (ie (1,3)-β-D-glucan), which directly interferes with the synthesis of (1,3)-β-D and destroys The skeletal structure and main antigenic components of the cell wall.

In the fluconazole control group, the cell membrane of *C. albicans* disappeared, and the nuclei and organelles dissolved and disappeared (Fig. 4e). Electron microscopy showed that cinnamic aldehyde acts on the cell wall of *Candida albicans* in vivo without affecting the cell membrane.

## Discussion

CA has antimicrobial properties, and determination of its minimum inhibitory concentration indicated that it is a powerful natural antimicrobial agent [21]. It has extensive pharmacological activity and can destroy the structure and functional integrity of fungal cell walls. The aldehyde group in the structure of CA is hydrophilic, allowing it to be easily absorbed by hydrophilic radicals on the fungal surface, thereby destroying the polysaccharide structure within the wall to penetrate the cell wall, affecting cell biosynthesis and inhibiting growth and reproduction [22]. (1,3)-β-D-Glucans mainly exist in the middle and outermost layers of the cell wall. They are the main antigen component of the cell wall, accounting for 80–90% of the dry cell weight. They form the skeletal structure of the cell wall with chitin and play an important role in maintaining cell integrity and structural stability. However, mammalian cells do not contain this component, so drugs targeting 1,3)-β-D-glucan are not toxic to mammals [23, 24]. Xie et al. [25] used electron microscopy and isotope labelling to show that CA acts on the cell wall of Aspergillus, destroying its structure, hindering the exchange of substances in and out of the cell, and affecting the absorption of substances and the synthesis of biological macromolecules, thereby inhibiting the growth and reproduction of this fungus. Wang et al., [26] established a series of Saccharomyces cerevisiae cell wall synthetase gene knockout strains (lKS1, GHS020 and GHS003) as models and found that CA can specifically inhibit the synthesis of fungal cell wall glucan and chitin. However, to date, the antifungal effects of CA have been studied only in vitro. This study found the following. (1) CA first acts on the fungal cell wall, affecting the synthesis of its main antigen component, (1,3)-β-D-glucan. This leads to the destruction of the skeleton of the cell wall and causes the cell wall to fall off and dissolve. The drug penetrates the cell body, eliminates the osmotic pressure inside and outside the cell, destroys organelles, and finally causes the fungus to rupture and die. This study verified the mechanism of action of CA in vitro and explained its antifungal effects of (1,3)-β-D-glucan on the fungal cell wall in vivo. (2) CA has strong fungicidal activity against invasive *Candida* infections in the body and can quickly kill pathogenic fungi, prolong the survival period of severely immunodeficient animals, increase the cure rate of invasive *Candida* infections, and reduce mortality, showing that CA has a unique antifungal effect via the cell wall. (3) Electron microscopy showed that the cell membrane of *C. albicans* treated with CA was still intact. This indicates that CA destroys only the fungal cell wall in the body without affecting the cell membrane. Currently, the main clinical drugs used to treat *C. albicans* infections are azoles, which target fungal cell membranes, have strong toxicity and side effects and are prone to drug resistance. Therefore, CA is an antifungal drug that acts on fungal cell walls for use in the treatment of severe fungal infections, especially *Candida* infections, and is unlikely to have toxic side effects on the human body.

## Conclusions

As invasive fungal infections have become more common and severe, they have also become an increasingly serious public health problem [27, 28]. Therefore, in the development of antifungal drugs, the development of glucan synthase inhibitors should be emphasized to reduce the toxicity and side effects of antifungal drugs and improve their effects in clinical treatment. CA can be extracted from cinnamon plants found in nature. CA acts directly on the cell wall of *C. albicans* and affects the synthesis of (1,3)-β-D-glucan but it does not affect the cell membrane or exhibit toxic side effects. This study provides a theoretical basis for the use of CA treatment against invasive *C. albicans* infection.

## Data Availability

The data in support of the results are available from the corresponding author on reasonable request.

## Abbreviations

CA: Cinnamaldehyde
*C. albicans*: *Candida albicans*
